# Understanding the Regulation Activities of Transposons in Driving the Variation and Evolution of Polyploid Plant Genome

**DOI:** 10.3390/plants14081160

**Published:** 2025-04-08

**Authors:** Yafang Xiao, Jianbo Wang

**Affiliations:** 1 State Key Laboratory of Hybrid Rice, College of Life Sciences, Wuhan University, Wuhan 430072, China; yafangxiao@whu.edu.cn; 2 School of Life Sciences, Guizhou Normal University, Guiyang 550025, China

**Keywords:** transposon, genome, polyploid, gene expression, epigenetic modification

## Abstract

Transposon is the main component of the eukaryotic genome, and more and more plant genome data show that transposons are diverse in regulating genome structure, variation, function and evolution, with different transposition mechanisms in the genome. Hybridization and polyploidy play an important role in promoting plant speciation and evolution, and recent studies have shown that polyploidy is usually accompanied by the expansion of transposons, which affect the genome size and structure of polyploid plants. Transposons can insert into genes and intergenic regions, resulting in great differences in the overall genome structure of closely related plant species, and it can also capture gene segments in the genome to increase the copy number of genes. In addition, transposons influence the epigenetic modification state of the genome and regulate the expression of the gene, while plant phenotype, biological and abiotic stress response are also regulated by transposons. Overall, transposons play an important role in the plant genome, especially polyploid plant genome, adaptation and evolution.

## 1. Introduction

Transposon, also known as transposon element (TE), or jumping gene, is a piece of DNA sequence that can move to different positions within the genome. The concept of transposon element was first proposed by McClintock in the late 1940s. She thought of them as chromosome control elements, and proposed an *Ac*/*Ds* regulatory system to regulate corn grain color [1], but this idea was not accepted for a long time, and transposon elements were once regarded as junk DNA or selfish genes [2,3]. However, since the beginning of this century, with the completion of the whole genome sequencing and assembly of various organisms, transposons have been confirmed to be the main components of the eukaryotic genome. More and more plant genome data show that transposon dynamics are more complex and diversified. Different transposition mechanisms can regulate not only the expression of adjacent genes in the genome, but also the entire genome, clarifying the importance of transposons in regulating genome structure, variation, function and evolution [4,5,6,7,8,9].

Hybridization and polyploidy have played an important role in promoting plant speciation and evolution, and most angiosperms have experienced at least one genome duplication event during their evolution [10,11,12,13,14]. A lot of important crops, such as wheat, soybean, cotton, peanut, oilseed rape and potato, are all polyploids produced by chromosome doubling recently or long ago. On the other hand, corn, rice and a few crops have long been considered as diploids, but some polyploidy events have also been identified in their evolution process based on the genome sequence analysis [15,16]. *Arabidopsis thaliana* is an important model plant with five chromosomes, and it is now confirmed that three or more rounds of genome-wide duplication have occurred during its evolution, owing to five to eight copies of many genes in its genome [17,18]. Hybridization and polyploidy generate a series of genetic and epigenetic changes, such as chromosome rearrangement, gene loss, DNA methylation and histone modification, while the duplicated genes can be lost, retained or maintained. Subfunctionalization and neofunctionalization can also occur [19,20,21]. Polyploids often exhibit different phenotypes from their diploid ancestors, which may help them adapt to new environments [22,23,24]. Genome-wide duplication through polyploidization has long been considered as a major contributor to genome evolution, and it is an important mechanism for speciation [25,26].

Polyploidy is usually accompanied by the amplification of transposons [4], which may increase the gene mutation rate and lead to changes in expression regulation due to the insertion of transposons in or near genes in the short term, and cause genome structure changes in the long term. On the other hand, genome-wide duplication allows it to buffer and cope with higher transposon activity [27]. Polyploidy and transposon amplification are not two completely independent mechanisms; conversely, the two phenomena interact to a large extent, reinforcing their potential to drive plant genome evolution together. Studies on the multiple factors that control transposon dynamics as well as polyploid genome characteristics will shed light on their evolutionary mechanisms [8]. This review covers the effects of transposons on diploid and polyploid plant genome size and structure, epigenetic modification, gene expression and their contribution to plant adaptation and evolution, with emphasis on polyploid plant genomes.

## 2. Type of Transposons in Plant Genome

Transposons are an important part of the plant genome. According to the sequence similarity and transposition mechanism of transposons, they are generally divided into Class I and Class II transposons. According to the different transposon-encoding transposase, they can also be divided into autonomous transposons and non-autonomous transposons [8,28,29].

### 2.1. Class I Transposons

Class I transposons, also known as retrotransposons, are first transcribed into mRNA in the transposition process, and then reverse-transcribed into cDNA by reverse transcriptase encoded by transposons. After transposition, the original DNA sequence is still retained, similar to the ‘copy-and-paste’ way, so it is considered to be the main contributor to the genome size. Based on the presence/absence of long terminal repeat (LTR) on both sides, it can be further divided into long LTR retrotransposons and non-LTR retrotransposons. The two superfamilies of LTR retrotransposons, *Copia* and *Gypsy*, are the most abundant in the plant genome, usually accounting for 90% of the total transposon content. They strongly influence genome size, and their copy number is closely related to genome size [8,30,31]. Non-LTR retrotransposons are mainly divided into two types: long interspersed nuclear elements (LINEs) and short interspersed nuclear elements (SINEs), usually with a simple repeat sequence at their 3’ end.

### 2.2. Class II Transposons

Class II transposons, also known as DNA transposons, can be mobilized by transposase cutting off the in situ DNA sequences and inserting them into a new site, similar to a ‘cut-and-paste’ approach [32]. In principle, this does not lead to an increase in the copy number of transposons, but it can be moderately increased. In general, class II transposons occupy a low proportion of transposons in plant genomes [33,34]. According to the transposition mode of class II transposons, it can be divided into DNA transposons in ‘cut-and-paste’ mode, *Helitron* superfamily in ‘rolling ring’ mode and miniature inverted repeat transposable elements (MITEs) [28,35], while some researchers classified *Helitron* superfamily transposons as the third type of transposon [36].

## 3. The Impact of Transposons on Genome Size and Structure

Plant transposons are the most variable part of the genome, as in most of eukaryotes. They often become the main and possibly the most proportional component of all plant genomic DNA, and their expansion/contraction can lead to large differences in the overall genome size and structure of even closely related plant species.

### 3.1. Transposons Contribute to the Variation of Plant Genome Size

Over the past two decades, remarkable progress has been made in sequencing the whole genome of plants, and the sequencing data indicated transposons are important dynamic components of the plant genome. In species with small genomes, such as Arabidopsis and *Brachypodium distachyon*, transposon abundance is 20–30% of the genome, but in species with larger genomes, such as maize and barley, it is about 85% of the genome [37]. It has also been found that the giant size of genomes in a fern (*Ceratopteris richardii*) and a gymnosperm (*Pinus tabuliformis*) is mainly attributable to the high transposable element contents [38,39]. Recently, the genomic assemble of an edible sweet lily variety, *Lilium davidii* var. *unicolor*, showed that its genome size reached 36.68 Gb, and transposons occupied 84.19% of the genome, mainly composed of LTR retrotransposons (LTR-RTs), accounting for 64.40% of the genome. The estimation of the insertion time indicated the sharply accelerated accumulation of LTR-RTs over the past 5 million years. These results indicate that transposon insertion events play an important role in the formation of giant lily genomes, besides from the direct influence of its whole genome duplication (WGD) [40]. A large number of polymorphic transposons were also found in the genomes of different cultivars in maize, and they played an important role in the variation of maize genome structure and gene number [41]. Transposons are likely to be the main contributor to the expansion and complexity of the genome by affecting its size, structure and function, and they have become an important factor driving the change and evolution of plant genomes [34,42,43,44].

### 3.2. Amplification and Elimination of Transposons in Plant Genomes

Although it has once been thought that the effects of transposon activity should irreversibly expand the genome, recent progress from a growing number of plant genome and resequencing datasets show that both amplification and elimination of transposons are highly dynamic processes in plant genome evolution [8,31,45]. There is a direct relationship between genome size, retrotransposon content and genome recombination rate. Even in the small genome of Arabidopsis, specific retrotransposon bursts have been found, and recent transposon bursts have occurred mainly in the centromere repeat region, which may be less harmful than inserting genes. Transposon-mediated dynamic processes accelerate the evolution of the Arabidopsis genome [30]. It was found that a member of MITEs, *mPing*, expanded from about 50 copies to about 1000 copies in four strains of rice, and most transposon insertion sites were located within 5 kb of the coding region. The rapid increase of the *mPing* copy number is a potential source of genetic diversity in rice and other inbreeding plants [46]. Further studies showed that the *Ping/mPing* family in the rice genome gradually achieves a high copy number by maintaining activity and avoiding silencing. An important reason for the successful expansion of the *Ping*/*mPing* family is that host recognition of transposons does not lead to the inactivation of transpotase [47,48]. Three LTR retrotransposons in the genome of wild rice *Oryza australiensis* expanded to 90,000 copies in 3 million years, leading to the double of the genome size [49].

### 3.3. Transposons Exhibit Their Complexity in Polyploid Plant Genomes

In addition to the effect of whole genome duplication, the expansion and elimination of transposons are also important factors affecting genome size in the polyploid plant genome, since the genome changes caused by interspecific hybridization and polyploid events can lead to transposon burst [27]. The ‘U’s triangle’ relationship of the *Brassica* (Brassicaceae) species consists of three diploid basic species and three allotetraploid species formed by hybridization and polyploidy among the diploid species [50]. The ratio of transposons in eight cultivars of *B. napus* (2n = 38, AACC) exceeded 55% of the genome [51], and the proportion of transposons in the genome of *B. juncea* (2n = 36, AABB) and *B. carinata* (2n = 34, BBCC) reached 50.36% and 58.34%, respectively [52,53], all higher than their diploid ancestors *B. rapa* (2n = 20, AA, 37.51%) and *B. oleracea* (2n = 18, CC, 38.80%) [54,55], but they are comparable to the proportion of transposons in the genome of diploid *B. nigra* (2n = 16, BB, 53.73%) [56].

In allotetraploid upland cotton (*Gossypium hirsutum*), it was observed that up to 64.80% of the genome sequences are transposons. The size of the A subgenome is about twice that of the D subgenome, and the transposon sequences of the A subgenome are also about twice that of the D subgenome. Further analysis showed that the ratio of transposons in the A and D subgenomes is consistent with that of their corresponding diploid ancestors. Transposons in the ancestral species are retained after allopolyploidization, while a small number of transposons in the D genome were amplified [57].

Transposon bursts also play an important role in the speciation of allotetraploid wild potato *Solanum stoloniferum*. It is found that transposons account for 64.86% and 65.21% of the A and B subgenomes of *S. stoloniferum*, respectively. The proportions of each transposon family in the A and B subgenomes are basically the same, and *Gypsy* LTR retrotransposons are the most abundant transposons. The transposon content and differentiation revealed recent transposon bursts and transposition events after hybridization and polyploidy, and its genome undergoes structural differentiation and mobilization of transposons, which is essential to stabilizing the genome equilibrium [58].

Hexaploid common wheat (*Triticum aestivum*, BBAADD) is one of the main cereal crops on the world. It is clear that diploid *Triticum urartu* (AA) and *Aegilops tauschii* (DD) are the ancestors of the A and D subgenomes, but the diploid ancestor of the B subgenome is controversial. Li et al. [59] assembled chromosomal-level genomes for five species of Sitopsis section, including *Ae. bicornis*, *Ae. longissima*, *Ae. searsii*, *Ae. sharonensis* and *Ae. speltoides*, and the results suggest that the donor of the B subgenome is a possibly extinct diploid species, distinct from all extant Sitopsis species, which diverged from the ancestor of the B-lineage to which extant *Ae. speltoides* belongs. The genomes of the five Sitopsis species differ in size, ranging from 4.11 Gb to 5.89 Gb, with high levels of repeats (85.99 to 89.81%), but there is a high degree of collinearity among them and the genomes or subgenomes of other species in the *Triticum–Aegilops* complex. The difference in genome size is mainly due to the expansion of transposons after the differentiation of independent species.

Broomcorn millet (*Panicum miliaceum*) is one of the earliest domesticated crops. However, its polyploid genome is still poorly understood due to the lack of extant diploid ancestors. Sun et al. [60] assembled a chromosome-level genome of allotetraploid broomcorn millet and found changes in subgenome size and the gene number caused by transposons. They divided the broomcorn millet genome into two subgenomes by using the genome sequence of *P. hallii*, a diploid relative of broomcorn millet. Contrastive analysis showed that the B subgenome was larger than the A subgenome due to biased accumulation of LTR retrotransposons in the ancestors of the B subgenome prior to polyploid formation. Notably, the accumulation of bias mutations led to more gene loss in the transposon-rich B subgenome.

In the allotetraploid *Brassica napus* genome, more than 910,000 transposons were identified, and most of them were *Helitron* and *Mutator* in class II transposons and *Gypsy* and *Copia* in class I transposons. The number of class II transposons was about three times that of class I transposons. Furthermore, it was observed that the ratio of the C subgenomic transposon sequence was higher than that of the A subgenome. This difference was mainly caused by the number of class II transposons, and more than 50% of the transposons were located within 8 kb of the flanks of genes, mainly within 2 kb. These transposons may regulate gene expression and thus regulate the adaptability of plants to the environment [61].

In some cases, polyploidy also leads to a reduction in transposon numbers, such as evidence found in the genome of allotetraploid tobacco (*Nicotiana tabacum*), in which the number of *Ty3-gypsy* transposon families is significantly reduced relative to their diploid ancestral genomes. Moreover, the elimination of transposons from the paternal *N. tomentosiformis* subgenome is more than that from the maternal *N. sylvestris* subgenome [62]. In general, transposons play an important role in the process of genome rebalancing after polyploid plant formation, but their acting mechanism and relative regulatory importance in different plants deserve further studies [27].

### 3.4. Transposons Affect Genome Structure and Variation

Transposons not only affect genome size, but also have important effects on genome structure and gene function. The polymorphisms produced by transposons can be integrated into host regulatory mechanisms and promote the formation of new cytological phenotypes. It was estimated that more than 1.2% of Arabidopsis protein-coding genes contain transposon-related sequences, especially the *Copia* and *CACTA* sequences [63]. Transposon insertion polymorphism (TIP) can cause structural variation of chromosomes, change gene sequence [33], regulate chromatin 3D folding structure [44] and produce more prominent genetic effects [34]. It is an important source of genetic variation related to plant genome evolution and distribution expansion [6,64,65]. Chen et al. [66] carried out a graph-based pan-genome analysis on 32 accessions of broomcorn millet (*Panicum miliaceum*) and further confirmed that the structural changes of the genome were closely related to transposons.

Although some transposon insertions are harmful, most of them are considered neutral or slightly harmful, and selection pressure will eliminate harmful insertions to limit their activity and potential mutation effects. Transposons pick out specific regions of the genome for integration [8], and create differentiation between gene-rich and transposon-rich regions within the plant genome [31]. Arabidopsis transposons and related tandem repeats were found early to be mainly distributed in heterochromatin regions [67], and in recent years many transposons have been found to target gene-rich regions, which may be a strategy to protect them from silencing by the host genome [8]. Transposon mutations produce new genes more frequently than currently thought, and they often coincide with critical periods of angiosperm origin and evolution [68]. Li et al. [69] completed the assembly of the gapless rice genome and identified more transpositions, which produced many repeat genes. These events could affect agronomic traits through dose effects or sub/neo-functionalization, and promote the evolution of these genes and rice genome.

Recently, it has also been found that Arabidopsis centromere sequences exhibit significant intraspecific and interspecific polymorphisms. The centromere-specific *ATHILA* transposons have been inserted into satellite sequence clusters, resulting in satellite repeat amplification to remove the transposons, which is consistent with the evolution cycle of repeat sequences. The rapid cycle of transposon insertion and satellite sequence amplification to remove the transposons drives the centromere evolution and ultimately speciation [70,71]. Moreover, Tsukahara et al. [72] assembled the complete centromere structures of two natural accessions of *Arabidopsis lyrate,* and revealed that both *Ty3* and *Ty1* LTR retrotransposons rapidly turn over within the centromeric tandem repeats of the *Arabidopsis* species. The *Ty1/Copia* element *Tal1* (*Transposon of Arabidopsis lyrata 1*) integrates *de novo* into regions occupied by CENH3 in *Arabidopsis thaliana*, resulting in the spread of *Tal1* integration regions. This study indicated the rapid transposon-mediated centromere structural change and evolution in plants.

*Nicotiana benthamiana* is a model organism widely used in plant research. Chen et al. [73] assembled the complete 2.85 Gb genome of allotetraploid *N. benthamiana*. Although the centromeres are widely dominated by *Ty3/Gypsy* retrotransposons, satellite-based centromeres are surprisingly common in *N. benthamiana*, with eleven of nineteen centromeres composed of megabase-scale satellite arrays. Moreover, the satellite-enriched and satellite-free centromeres are extensively invaded by distinct *Gypsy* retrotransposons, indicating their crucial roles in centromere function. Finally, they proposed that satellite expansion, retrotransposon enrichment and neocentromere formation play important roles in *N. benthamiana* centromere evolution.

### 3.5. Transposon Insertion Affects Chromatin Accessibility

In the nucleus, chromatin is highly organized and consists of DNA, proteins and RNA. Chromatin accessibility is the accessibility of the DNA regions of chromatin to transcription factors, chromatin modifying enzymes and other regulatory factors. The accessibility of chromatin directly affects the binding by regulatory factors, and it plays a key role in gene expression regulation. Chromatin accessibility is shaped by chromatin structure and epigenetic modification, and transposons also affect chromatin accessibility. There is evidence that many accessible chromatin regions (ACRs) exist in transposons, and in some cases such sequence distribution features have regulatory activity [74]. In the genome of maize B73 variety, hundreds of ACRs were identified to be disrupted by transposon insertion, and thousands of ACRs were located within transposons. Transposon insertion into ACR can alter chromatin accessibility, while many ACRs with transposon insertion show evidence of functional enrichment, suggesting that transposons may influence maize gene expression by regulating chromatin accessibility [75]. By using the ATAC-seq method to analyze the chromatin accessibility of the leaves and petals of the diploid, neo-autotetraploid and evolved autotetraploid *Arabidopsis arenosa*, it was found that the chromatin accessibility variation across different cytotypes is closely related to the number of nearby transposons [76].

## 4. Epigenetic Modification of Transposons

Epigenetic studies do not rely on the original DNA sequence but on DNA and chromatin modification of traits and gene expression changes. Epigenetic traits are heritable and reversible, often associated with DNA methylation, chromatin modification, and/or RNA-mediated mechanisms. DNA and chromatin modifications are inherited to different cell types and/or offspring through mitosis and meiosis, known as epigenetic memory. Both external (environmental) and internal (genomic) stresses and signals can induce heritable epigenetic variations in plants, and most internally induced epigenetic variations are relatively stable and can be used to improve crop yield and stress resistance [77].

Epigenetic regulation is a key regulatory mechanism of gene expression. Plant transposons are usually silenced by specific epigenetic mechanisms owing to their sequence characteristics and genome location. Post-transcriptional silencing and transcriptional silencing of transposons are related to DNA methylation, histone modification and small RNA, etc., which is a complex systematic regulation process. Different epigenetic modification pathways play various roles in transcription, duplication and recombination regulation of transposons, and epigenetic changes that respond rapidly to environmental conditions may evolve faster than genetic changes [7]. The specific regulation of different transposons and their different roles in chromosome structure and gene expression regulation are determined by their positions on chromosomes, such as near genes, within genes, near centromeres or within centromeres. Their localization on chromosomes is the primary rule that determines the specific regulation of each transposon [78].

### 4.1. DNA Methylation of Transposons

DNA methylation is essential for the development of plants and animals. In plants, methylation occurs via different pathways at CG, CHG and CHH (H = A, C, or T) sites, and the methylation of transposon sequences also includes CG, CHG and CHH contexts, with silent transposons typically only hypermethylated in CG and CHG contexts, while CHH methylation levels are usually much lower. DNA methylation in the coding region of a gene is often associated with moderate to high levels of gene expression, while methylation in the promoter region usually suppresses or silences the relevant gene [79]. In addition, CG methylation maintains the stability of centromere and chromatin, while CHG and CHH methylation occurring in transposons adjacent to genes often leads to gene and transposon silencing or heterochromatin formation [80,81]. Silent transposons may affect gene function and expression, especially when it is close to a gene [82]. Whole genome sequencing and DNA methylation analysis of hundreds of natural Arabidopsis accessions have shown that transposons exhibit significant intraspecific genetic and epigenetic variation, and the genetic variation is often the basis of epigenetic variation. The epigenetic modification and selection forces together determine the extent to which transposons may affect genome evolution, which plays a crucial role in transposon–host coevolution [83].

In order to explore the effect of whole genome duplication (WGD) rather than hybridization on DNA methylation, Zhang et al. [84] conducted a comparative analysis on DNA methylation and gene expression of autotetraploid rice and its parents, and observed the universal transposon methylation in the autotetraploid genome. In particular, the CHG and CHH contexts of DNA transposons are hypermethylated, which is accompanied by an increase in the abundance of 24-nt small RNA, indicating that the RNA-directed DNA methylation (RdDM) pathway may take place. Their results show that class II transposon hypermethylation inhibits the expression of neighboring genes. In summary, this study shows that polyploidization brings about transposon methylation changes that will affect gene expression, which is an effective way for the new autotetraploid to respond to the new genomic environment.

In a further study on autotetraploid rice [85], it was found that there were no significant changes in CG and CHG methylation levels in the tetraploid rice genome compared with diploid rice, but CHH methylation levels were lower than that of diploid rice, and polyploidy induced hypomethylation of CHH. It was also found that the methylation level of diploid rice did not change under salt stress, but the CHH methylation level of protein-coding genes and transposons of tetraploid rice increased, indicating that salt stress promoted hypermethylation of tetraploid rice. Under environmental stress, transposons tend to be activated and transposed to accessible chromatin regions (including regions with low DNA methylation). Then, the expression of activated transposons induces DNA hypermethylation, thereby inhibiting the expression of transposon-related genes. This feedback regulation of transposons and stress-responsive genes gives polyploid plants an evolutionary advantage to adapt to the environment.

### 4.2. Histone Modification of Transposons

Histone demethylation is an important mechanism to control retrotransposon activity. Cui et al. [86] found that rice JMJ703 protein is an active H3K4-specific demethylase, and the impaired JMJ703 activity leads to the increase of H3K4me3 level, which leads to the activation of two non-LTR retrotransposon families. However, the loss of JMJ703 did not affect transposons previously found to be silenced by other epigenetic pathways, suggesting that active histone modifications are indeed involved in transposon silencing, but that different types of transposons may be regulated by different epigenetic pathways. Between the A and C subgenomes of *Brassica napus*, the differences in the transposon methylation and histone modification status were observed, and the epigenetic modification gradually recovered to a level close to that of the ancestral species during the formation and evolution of allotetraploid *B. napus* [61]. On the other hand, some transposon silencing events are associated with H3K27me3 modification, indicating an alternative mode of transposon silencing instead of DNA methylation in flowering plants, which suggests a dynamic switching between the two epigenetic marks at species level of plants [87].

### 4.3. The Effect of Non-Coding RNA on Transposon Activity

Non-coding RNAs also exert dynamic effects on genomic transposons. The 24-nt siRNA formed by transposon-derived small interfering RNA (siRNA) loci is the main component of endogenous siRNA in plants [88]. MicroRNAs (miRNAs) regulate siRNA synthesis in Arabidopsis after the activation of transposons, preventing transposons to evade long-term heterochromatic silencing [89], and the non-additively expressed siRNA clusters resulted in lower activity of the transposons through DNA methylation in the *Brassica napus* hybrid [90] and cotton hybrid [91]. Hollister et al. [92] found that the transposon copy number of *Arabidopsis lyrata* was two to three times higher than that of *A. thaliana*, while the effect of siRNA-directed DNA methylation was lower in *A. lyrata*, which may reveal the reason for the differential expansion of transposons between species.

In order to reveal the mechanism of subgenome dominance, Cheng et al. [93] studied the relationships among gene expression, transposon distribution and small RNA targeting, and found the biased distribution of transposons among subgenomes and the targeting of 24-nt small RNAs together cause the dominant expression at a subgenome level. In addition, transposons had highly conserved copies at mildew resistance loci, and *Mariner*-derived miRNA played an important role in wheat immunity [94]. In rice, miR812w derived from *Stowaway* type transposons played an important role in rice blast resistance [95].

Recently, long non-coding RNA (lncRNA) is also found to be involved in the regulation of genomic transposon activity, especially the intergenic lncRNA (lincRNA) transposon content which affects transposon expression and epigenetic modification, indicating that cells fight the spread of transposons through a complex system that inactivates them and prevents them from damaging the genome [96].

## 5. Capture of Gene by Transposons in the Genome

Transposons not only affect genome size and evolution, but also have a significant impact on the occurrence and evolution of new genes in plants. A distinguishing feature of many transposons is their ability to capture and move genes or gene fragments, a process known as transduplication [6].

### 5.1. Gene Capture by MULE and Helitron Transposons

Mutator-like transposable elements (MULEs) have been found to be capable of capturing complete or partial gene segments and transpose them to new locations in the genome in maize, rice and Arabidopsis, potentially forming new gene structures, and they are called Pack-MULEs [7]. The 3000 Pack-MULEs capture more than 1000 gene segments in rice, which typically contain fragments from multiple genomic loci that fuse to form new open reading frames. While many of these gene segments may be nonfunctional pseudogenes, some of them are expressed as chimeric transcripts that can become retrogenes. About a fifth of Pack-MULEs contain segments from multiple genomic loci, suggesting that Pack-MULEs have the potential to create new genes by duplicating, rearranging and fusing different genome sequences [97]. Juretic et al. [98] also identified 8274 MULEs with complete terminals and target duplications in rice, among which 1337 contained host duplicate gene segments. Through analysis of the transcribed duplicate gene segments, it was proved that almost all cases had pseudogene characteristics, including fragmented conserved protein domains, frameshift mutations and premature termination codons.

Based on genome-wide comparative analysis, Wang et al. [99] studied the occurrence process and evolutionary mechanism of MULEs-derived genes in 10 species of genus *Oryza*, and observed that MULEs-derived genes mainly came from GC-rich donor sequences. MULEs preferentially captured donor sequences from genomic regions with low methylation levels and high recombination rates, and the methylation levels of the interior and inverted end repeat regions of these elements were regulated by the 24-nt siRNA-directed methylation pathway. DNA methylation may be the main mechanism promoting the occurrence, survival and expression of MULEs-derived genes; thus, it can promote the formation of new genes in the process of plant genome evolution.

Unlike ordinary DNA transposons, *Helitron* transposons employ a replicative rolling-circle mechanism that leaves the original template unaltered, and it uses the ‘peel-and-paste’ mechanism to achieve gene capture, leading to an increase in gene copies and promoting genome evolution. In the polyploid *Brassica napus* (AACC) genome, the *Helitron* superfamily has the highest number of transposons [61]. In a recent study, 3156 capture events and 326 donor genes by *Helitrons* were identified. The captured donor genes were related to the number, length and location of their exons, while the gene-capturing *Helitrons* carrying donor gene fragments were evenly distributed on the genome. More than half of captured genes were involved in the construction of pseudogenes, becoming the reserve for polyploid genome evolution. Moreover, the siRNAs targeting transposons may act on the donor genes due to siRNA crosstalk, and the gene expression levels decreased accompanying DNA methylation levels which increased. The genome sought a balance between sacrificing donor gene expression and silencing transposons, while epigenetic modifications may temporarily relax the control of gene capture during allopolyploidization process [100].

### 5.2. Genomic Conflict Caused by Gene Capture

Gene capture meets the evolutionary interest of transposons, because it blurs the boundary line between the host and transposon by combining transposon and host sequences, which will increase the cost for the host to effectively silence these transposons. This idea put forward a genomic conflict model, in which transposons capture gene fragments, while the host produces an siRNA-mediated response to the transposon [6]. The siRNA encoded by the captured fragment in the transposon can also target the corresponding region of the ‘donor’ gene, and this targeting will lead to DNA methylation and subsequent silencing. As a result, the host response to the capture behavior of the transposon can transact on the donor gene at the same time. In this case, transcriptional silencing of transposons may have collateral effects on the donor gene; i.e., silencing transposons comes at the cost of silencing the gene. However, if the gene is important, natural selection may maintain function by regulating the silencing response, which may also favor transposons. Notably, the conflict model made testable predictions that: (i) Donor genes have characteristics of *trans*-epigenetic effects, including increased siRNA targeting and consequent methylation effects. (ii) Natural selection may limit the *trans*-epigenetic effects of important genes relative to less functionally important genes. (iii) Transposons benefit from capture by reducing host response. This model was tested by analyzing *Helitron*, *Pack-MULEs*, and *Sirevirus LTR* retrotransposons in maize genomes through identifying 1263 transposons containing exon fragments from 1629 donor genes. Consistent with epigenetic conflicts, transposed donor genes transcribed more siRNA than non-captured genes, and siRNA was associated with CHH methylation, suggesting that transposon capture triggered intra-genomic conflict, which may not affect the function of important genes but may lead to pseudogenization of other genes [82].

### 5.3. Transposons Take Part in miRNA Gene Amplification in Polyploid Plant Genome

Some miRNA families contain multiple members that produce the same or highly similar mature miRNAs in plants, but the mechanism for miRNA gene family expansion is unclear, and it has been proposed that it may be achieved through tandem and/or fragment duplication. Shen et al. [101] analyzed the gain and loss of members of the *MIR482/2118* superfamily in two tetraploid cotton, *Gossypium hirsutum* and *G. barbadense*, and their extant diploid ancestors, *G. arboreum* and *G. raimondii*. The results showed that the *MIR482/2118d* gene was significantly amplified in *G. hirsutum*, *G. barbadense* and *G. raimondii*, but not in *G. arboreum*. Based on the analysis of the flanking sequences of these *miR482/2118* loci, it was found that the amplification of *MIR482/2118d* and its derivatives was due to the initial capture of *MIR482/2118d* by class II DNA transposons (*PIF/Harbinger* superfamily) in *G. raimondii* prior to the polyploidization event. Subsequently, they transposed to new locations in the genomes of *G. hirsutum*, *G. barbadense* and *G. raimondii*. All homoeologous *MIR482/2118* loci of two diploids are retained in both tetraploids, but mutations in *MIR482/2118* have been observed in all of the four species, suggesting dynamic and coordinated evolution of the *MIR482/2118* and its target genes. This study showed that gene capture by transposon is one of the important methods for miRNA gene family expansion.

## 6. The Impact of Transposons on Gene Expression Regulation

In addition to the overall impact on the size and structure of plant genomes, transposons can also participate in the gene expression regulation network in a *cis*- or *trans*-acting mode at the transcriptional or post-transcriptional level, which confirms the theory of transposable elements as ‘control elements’ proposed by McClintock [36,90,102]. Transposon insertion is usually associated with alternative transcription start sites or transcription end sites of the genes, and the epigenetic state of intragenic transposons affects transcription and alternative poly(A) signals of transcripts, resulting in different isoforms [103].

Aimed at understanding the amplification of the *mPing* transposon in rice, Naito et al. [104] analyzed and evaluated the effects of 1664 insertion sites of *mPing* transposons on the expression of 710 genes, and found that most of the transposon insertions caused up-regulation of gene transcription or had no detectable effects. This modest effect reflects that *mPing*’s preference for inserting into 5’ flanking sequences and the avoidance of exons, providing evidence for previously proposed models for transposons and other repeats involved in genome recombination and gene expression regulation.

Polyploidy provides evolutionary and morphological novelty to many plants. However, the role of genomic dose and composition in regulating gene expression remains poorly understood. Kashkush et al. [105] first discovered that LTR retrotransposons activate adjacent gene expression in polyploid plants. Their studies showed that *Wis 2-1A* retrotransposons were statically expressed in newly synthesized allopolyploid wheat, and the transcribed transcripts were involved in regulating the silencing or activation of the corresponding genes. It is proved again that transposons are potential regulatory factors.

In order to investigate the effects of ploidy and hybridization on gene expression, Shi et al. [106] resynthesized a series of *Arabidopsis* tetraploids containing zero to four copies of the *Arabidopsis thaliana* and *Arabidopsis arenosa* genomes. They found that allelic dosage-dependent genes tended to be further away from transposons than dosage-independent genes, and that fewer transposons near dosage-dependent genes indicated that these transposons were harmful and had been eliminated by purifying selection. Thus, transposons shape the diversity of gene expression in polyploid plants, and dosage-dependent expression can maintain growth and developmental stability, while dosage-independent expression can promote functional differentiation (subfunctionalization and/or neofunctionalization) between homoeologous genes during polyploidy evolution.

Transposons are also involved in the regulation of subgenomic dominance in polyploid plants. Subgenomic dominance refers to the superiority of gene expression in one subgenome over another. Transposons have been found to be involved in the regulation of subgenomic dominance in many polyploid and paleopolyploid plants. Whole genome duplication (WGD) occurs repeatedly in angiosperms. In paleo-tetraploids, the two subgenomes are distinguishable because one subgenome, the dominant subgenome, tends to have more genes than the other. In addition, in the retained gene pairs, genes on the dominant subgenome tend to be more highly expressed than recessive homologous genes. Woodhouse et al. [107] found that transposon-derived 24-nt siRNA in *Arabidopsis thaliana* and *Brassica rapa* preferentially targeted the upstream region of genes in the recessive subgenome and affected their gene expression levels. This result suggested that the successful paleo-tetraploid began with extensive hybridization between two strains, and each hybrid has a different trade-off between transposon silencing and negative position effects of gene expression. After the mixed extensive hybridization-neo-tetraploid formation period, genes acquire a new expression balance based on parental transposon differences, and transposon silencing can act as a *cis*-regulator of transcription. However, a study on the resynthesized allotetrapolyploid offspring from *Brassica rapa* and *B. oleracea* hybridization and chromosome doubling did not show the negative correlation between transposon silencing and gene expression in the resynthesized allopolyploids, reflecting the complexity of subgenomic dominance. The new polyploids may exist as the form of genomic chimera. At this stage, the two subgenomes may function independently, and then subgenomic dominance occurs during genome evolution (diploidization), just as subgenomic dominance occurs in paleo-polyploidy [108]. Therefore, the regulatory effects and mechanisms of transposons on gene expression are varied in different stages of polyploid formation and evolution, and further studies need to be carried out in more plants to further reveal the evolution of polyploid genomes.

The success of allohexaploid common wheat as a major crop in the world is largely due to the genomic diversity and gene redundancy resulting from the genome-merging of different species, which raises the question of how subgenome-divergent and convergent transcription are coordinated in a single cell. Zhang et al. [109] conducted a genome-wide transcription factor binding-sites (TFBSs) analysis, and assembled a common wheat regulatory network. They found that a large portion of subgenome-divergent TFBSs is derived from differential expansions of specific transposons in diploid ancestors, which contributes to subgenome-divergent transcription, while subgenomic convergent transcription is associated with balanced TFBSs generated from transposon expansions before diploid divergence. Differential evolutionary selection of paleo- and neo-transposons promoted the regulation of subgenome-convergent and -divergent regulation in common wheat, highlighting the influence of transposon plasticity on polyploid transcriptional plasticity.

In general, transposons are a rich source of non-coding regulatory elements whose inherent properties and their conflict relationship with the host promote their regulatory functions in different genomes [35]. Recent advances in genomics and epigenomics enable us to analyze the distribution of transposons in the genome and their regulatory functions of gene expression, and have in-depth understanding on plant development and evolution [9].

## 7. The Regulation of Plant Phenotypes by Transposons

The *Ac*-*Ds* transposon system regulates the grain color of maize, a transposon first proposed by McClintock [4], so the influence of transposons on plant phenotype has always attracted attention. Recent advances in genomics and phenomics have made it possible to precisely link genotypes to phenotypes [6].

### 7.1. Transposon Insertion Regulates Maize Phenotype

Transposon insertion can up-regulate or down-regulate gene expression and affect plant phenotype. A classic example is that *Mutator* transposon insertion into the intron of the maize *knotted1* (*kn1*) gene affects the phenotype [110]. A series of dominant mutations at the maize *kn1* locus are dominant mutations that affect leaf development, and another 10 mutant alleles in some maize lines with *Mutator* transposons were recorded in this study. Nine of these alleles had *Mu1* or *Mu8* transposon insertions within 310 bp of intron 3 of the *kn1* gene, and these insertions affected *kn1* gene expression and the phenotypes of node and leaf of maize. Salvi et al. [111] found MITE transposon insertion at 70 kb upstream of *Ap2*-like transcription factor gene, which could regulate the flowering time of maize. This was the first proof that transposon insertion into conserved non-coding sequences could affect important agronomic traits, even with transposon insertions far away from the genes. The *stiff* gene, a major quantitative trait locus for maize stem strength, was also identified to be regulated by transposon. There is a transposon insertion in the promoter of the *stiff* gene, which inhibits the transcription of this gene, resulting in increased cellulose and lignin content in the cell wall, thereby increasing stem strength [112].

### 7.2. Rice Agronomic Traits Affected by Transposon

Although many transposon-related mutations have been identified, there is very limited evidence that transposons regulate crop yield and other agronomic traits. Tillering is the key trait determining plant structure and yield in rice. Xu et al. [113] found that 24-nt siRNA produced by *OsNRPD1a* and *OsNRPD1b* (two orthologous genes of the largest subunit of RNA polymerase IV) can induce DNA methylation of transposons (including MITEs). Transposon methylation positively or negatively regulates the expression of many genes, among which *OsMIR156d* and *OsMIR156j* genes that promote tillering in rice are inhibited by CHH methylation in two MITEs in the promoter region, whereas *D14* genes that inhibit tillering in rice are activated by CHH methylation on one MITE downstream of them. Their results reveal the regulation of transposon RdDM methylation on rice tillering, and it also provides a potential target for improving agronomic traits through epigenome editing. Moreover, a gene named *PANICLE NUMBER AND GRAIN SIZE* (*PANDA*) was identified to be derived from rice *Harbinger* transposons, which was neo-functionalized to regulate panicle number and grain size. The research provides new understanding of the epigenetic control of *Harbinger* transposon-derived genes on rice yield traits and provide potential applications in improving rice yield [114].

Based on 247 chromosome level genomes of rice, a pan-transposon map of cultivated and wild Asian rice was developed, which detected 177,084 high-quality transposon variants, and found that transposons are a source of phenotypic variation during rice domestication and differentiation. The results highlight the contribution of transposons to rice domestication, differentiation and agronomic traits, and the potential application of high-quality pan-transposon maps in cultivated Asian rice in gene cloning and molecular breeding [115].

### 7.3. Transposons Regulate Plant Fruit Morphotype

The color of the grape skin is determined by the accumulation of red plant pigments called anthocyanins. White grape cultivars are thought to have arisen from red grape cultivars through independent mutations, but the molecular mechanism for these color mutations is unknown. *Myb*-related genes, such as *VlmybA1-1*, *VlmybA1-2* and *VlmybA2*, regulate anthocyanin biosynthesis in black-skinned cultivars of *Vitis labruscana*. Kobayashi et al. [116] found that *Gret1* retrotransposon is inserted into the *VvmybA1* gene in cultivars of the white grape of *V. vinifera*, which affects the expression of this gene and the color of grape skin. This retrotransposon-induced mutation is associated with the loss of pigmentation.

Transposons play important roles in the color regulation of blood orange (*Citrus sinensis*). Sicilian blood orange is produced by inserting a *Copia*-like retrotransposon near the *Ruby* gene, which is a *MYB* transcription factor gene of anthocyanin synthesis. Retrotransposons control the expression of the *Ruby* gene, and cold temperature dependence reflects the induction of the retroelement by stress [117]. In addition, Tarocco blood orange is derived from the insertion of a retrotransposon into the promoter of the *CsRuby1* gene, which contains *cis*-elements that are sensitive to low temperature, and the color formation of its fruit is also dependent on low temperature [118]. These studies suggest that the transposition and recombination of retrotransposons may be important sources of citrus variation.

In flowering plants, hermaphroditism plants are the dominant sexual morph, and little is known about the emergence of unisexual plants. Using melon (*Cucumis melo*) as a model system to explore the driving mechanism of sexual forms, it was revealed that the *Ethylene Insensitive 2* (*CmEIN2*) gene plays a dual role in sex determination and fruit morphogenesis. A spontaneous mutant exhibits the transition from a bisexual to unisexual male flower, and *Harbinger* transposons were activated in the mutant, while the expression of the *CmEIN2* gene was inhibited [119].

Furthermore, Zhang et al. [120] discovered fruit color variation in a melon (*Cucumis melo*) variety characterized by uneven and mottled dark green streaks and spots on the white fruit, called ‘Shooting Star’ (SS). The dark green spots or streaks in the pericarp are the result of chlorophyll accumulation in the white pericarp. Further investigation revealed that the SS phenotype is controlled by a single dominant gene, *CmAPRR2*, in which a *hAT*-like transposon is inserted, which is repeatedly excised in the *CmAPRR2* gene in the pericarp tissue. Activation of the expression of the *CmAPRR2* gene promoted the accumulation of chlorophyll, resulting in dark green in the pericarp, and finally formed the SS pericarp phenotype. Therefore, the SS phenotype was thought to be caused by the repeated excision of *hAT*-like transposons in the *CmAPRR2* gene.

Based on the high-quality genomes of eleven *Capsicum* species (including wild and domesticated pepper species), it is suggested that the expanded and variable genome sizes were attributed to differential transposable element accumulations in various species, and transposon shaped 3D chromatin architecture and caused mutations related to pepper traits such as fruit orientation and color [121]. In addition, tomato fruit shows abundant morphological polymorphism. *SUN* gene duplication mediated by *Rider* LTR retrotransposon causes gene expression changes, resulting in the formation of elongated tomato fruit, which indicate that retrotransposon-mediated retro-transposition can lead to the functional reprogramming of gene expression [122].

### 7.4. The Effect of Transposon Methylation on Cotton Fiber Development

Cotton (*Gossypium hirsutum*, AADD) is an allotetraploid consisting of two ancestral genomes. Each cotton fiber is a cell that rapidly elongates from the epidermis of the ovule, but the molecular basis of this developmental transformation is unknown. Song et al. [123] found that fiber cells in ovule have additional RdDM-independent pathway CHH hypermethylation, which inhibits the expression of transposons and nearby genes including fiber-related genes. RdDM-dependent methylation of promoters and RdDM-independent methylation of transposons and their nearby genes may act as a double-lock feedback mechanism to regulate gene and transposon expression, enabling epidermal cells to transform into fiber cells during ovule and seed development.

### 7.5. The Effect of Transposons on the Phenotype of Other Plants

Pan-genomic analysis of the 524 cultivars of *Brassica rapa* found that 23% of genes had transposon insertion polymorphisms (TIPs) relative to the reference genome of the ‘Chiifu’ cultivar. These TIPs widely affected the structure and function of trait-related genes, thus playing an important role in the domestication process of *B. rapa* with different morphotypes [124]. Moreover, a *hAT*-*Ac* transposon was located in the promoter region of the *MYB* transcription factor gene, *MYB12*, and the specific expression of *MYB12* mediated by this transposon is crucial for the formation of yellow tepals in lotus, which provided a molecular basis for the breeding of yellow lotus [125]. Generally, the above results indicate that transposons contribute to the formation of complex plant traits (including many important agronomic traits), but the molecular regulatory mechanisms are not well understood, and this is worth further research.

## 8. The Regulatory Role of Transposons in Plant Response to Stress

Many studies on transposons have shown that they may be highly sensitive to a variety of biological and abiotic stresses, including salt, low temperature, and heat stress, as well as stress caused by fungal, bacterial, and viral infections. In some stress situations, the insertion of transposons upstream of host genes can make these genes respond to stress, and transposon activity can increase the total number of stress-inducing genes and make the genome respond to stress, so as to increase the ability of plants to resist stress [6,126,127,128].

### 8.1. Transposons Response to Abiotic Stress

In Arabidopsis seedlings under heat stress, a type of Copia retrotransposon called ONSEN not only has transcriptional activity, but also synthesizes extra-chromosomal DNA copies. Naturally and experimentally induced ONSEN insertion variants confer thermal responsiveness on nearby genes, so transposon migration may generate new stress-response gene regulatory networks [129]. In addition to ONSEN, the expression of Copia-35 retrotransposons is also induced by heat stress in Arabidopsis, and the activation of ONSEN and Copia-35 and other transposons can up-regulate the expression of APUM9 and other flanking genes, which may affect the flowering time [130].

Evaluation of the expression of transposon families in the leaf tissue of three inbred lines of maize (*Zea mays*) under heat or cold stress found no evidence of whole-genome activation of transposons; however, some specific transposon families only produce transcripts under stress. In different genotypes, transposon families showed significant differences in stress response expression. When transposons were shared in two genomes, full methylation inhibited the expression of one of the genotypes. This study provides insights into the regulation of transposon expression under stress conditions, and highlights the impact of chromatin variants on different transposon families or genotypes [131].

Polyploid plants often show enhanced adaptability in diverse and extreme environments, but their molecular basis remains unclear. Wang et al. [85] found that salt tolerance in tetraploid rice is enhanced by reducing sodium intake and is associated with epigenetic regulation of jasmonic acid (JA)-related genes, and is usually associated with adjacent transposons of genes. Under salt stress, stress response genes (including JA pathway-related genes) are induced and expressed more quickly in tetraploid rice than in diploid rice. The elevated expression of stress-response genes in tetraploid rice can induce hypermethylation and expression inhibition of nearby transposons. In rapid and intense stress responses, there is a feedback relationship between polyploid-induced demethylation and stress-induced hypermethylation to inhibit nearby transposons and/or transposon-related stress-response genes.

### 8.2. Transposons Response to Biotic Stress

Transposons also play an important role in response to biological stress. For example, some transposons are demethylated and transcriptionally reactivated during the bacteria infection process defense of Arabidopsis, and this effect is associated with down-regulated expression of key transcriptional silencing factor genes, partly dependent on active demethylation processes. DNA demethylation restricts the proliferation of the bacterial pathogen *Pseudomonas syringae* in the leaf vascular bundle, since some immune response genes containing repeats in the promoter region are negatively regulated by DNA methylation [132].

Plants synthesize many specific metabolites through biosynthesis-related gene duplication and functional specialization. However, it is not clear how duplicated genes are integrated into existing regulatory networks. In Arabidopsis, this exaptation occurs by inserting the LINE retrotransposon *EPCOT3* into the enhancer. The repeating gene *CYP82C2* has been recruited into the WRKY33 regulon and indole-3-carbonylnitrile (ICN) biosynthetic pathway. The presence of the WRKY33 binding site on *EPCOT3* enhances the neofunctionalization of the *CYP82C2* gene and promotes the evolution of this gene involved in the synthesis of the defense metabolite 4-hydroxyl ICN. These results bring us to a deeper understanding of how duplicated genes and transposons work together to promote chemical diversity and pathogen defense [133].

## 9. The Role of Transposons in Plant Adaptation and Evolution

The amplification of transposon can rapidly produce genetic variants that, in some cases, improve an organism’s ability to adapt to its environment. For example, the insertion of transposons may affect genes associated with flowering time and thus affect the flowering time of the plant, making it better able to adapt to a particular ecological environment. On the other hand, although the amplification of transposons can bring adaptive evolutionary advantages, it can also pose a threat to the stability of the genome. Excessive transposon activity may lead to genomic instability and may even affect the viability of organisms.

The mobilization of transposons is a potent source of mutations, and several transposons of plants respond to external cues, supporting the hypothesis that natural transposition can create environmentally sensitive alleles for adaptation. For example, Raingeval et al. [134] reported on the characterization of a retrotransposon *ONSEN* insertion within the first intron of the Arabidopsis floral-repressor gene *FLOWERING LOCUS C (FLC)* and its role for adaptation. The insertion mutation enhances the environmental sensitivity of *FLC* by affecting the balance between coding and non-coding transcripts in response to stress, thus expediting flowering. The stress-sensitive allele of *FLC* has spread across populations subjected to recurrent chemical weeding, and retrotransposon-driven acceleration of the life cycle represents a rapid response to herbicide application. This study provides a compelling example of a transposon-driven environmentally sensitive allele that confers an adaptive response in nature.

Based on large-scale pan-genome sequencing data, a lot of transposon-induced structural variants were detected by using 811 high-quality genomes from 119 plant species, revealing a mutual evolutionary relationship between transposons and host genomes. Moreover, transposons cause genetic variation by inducing the duplication of host genes and inserting into regulatory regions [135]. These transposon dynamics not only reveal evolutionary features linked to transposition activity but also highlight the role of transposons in plant adaptation.

The increase in the activity of transposons can lead to genetic diversification within natural populations, resulting in new adaptations, fixing the variants brought about by natural selection in subsequent selection, and the transposons are no longer viewed as negligible components of the genome, but as potential contributors to adaptive evolution. The molecular mechanisms by which transposons produce new genetic variants are diverse, and Schrader and Schmitz [136] divided them into five different categories: (i) Genomic transposon protein-coding genes can be domesticated and co-opted by providing the original codon structure to express a novel and adaptive host trait. (ii) After insertion of a transposon into an intron of a host gene, parts of the transposon can be co-opted by including a novel, emerging the transposon exon to an existing protein-coding gene in a process called exonization. (iii) Transposon transposition into the proximity of genes can affect their regulatory environment and thus transcription. (iv) By the activity of the transposition machinery of Class I long interspersed elements (LINE1), the arising gene transcripts can be reverse-transcribed and inserted into the genome as intronless retrocopies. (v) The presence of paralogous copies of transposons in a genome can provide the substrate for aberrant transposition and ectopic recombination leading to novel structural rearrangements and genomic plasticity. On the other hand, transposons have the potential roles to cause reproductive isolation across a diversity of taxa [137], and increased activity of transposons might take part in hybrid speciation in animals and plants [138]. Polyploidy is usually accompanied by the expansion of transposons, which may increase the gene mutation rate and change expression regulation due to the insertion of transposons in or near genes in the short term, and cause changes in genome structure in the long term. Polyploidy and transposon variation interact with each other and jointly drive the evolution of plant genomes [8]. Allohexaploid wheat transposons accounted for 85% of the genome, and the transposon proportions of the subgenomes A, B and D were 86%, 85% and 83%, respectively. No transposon burst was observed in the polyploid wheat genome, and the characteristics of transposon family proportions between subgenomes and transposon enrichment near genes were accidentally preserved, which strongly suggests that transposons may function at the structural level under selection pressure [139]. In *Capsella bursa-pastoris*, although allopolyploidy has been observed to have a significant effect on genome-wide transposon abundance, significant transposon amplification was observed in gene-enriched regions, suggesting that relaxed selection may affect genomic transposons [140]. Diversification through subgenomic transposon exchange has also been found in allopolyploid cotton genomes, thus balancing genome size, evolutionary rate heterogeneity and positive selection among homologous genes, promoting polyploid gene family diversification and homologous gene expression differentiation [141].

During evolution, successful allopolyploids must overcome the ‘genome shock’ between ancestor species, but the underlying process remains elusive. Transposon-induced epigenetic gene and gene expression changes may allow plants to overcome genomic dose effects, potentially contributing to rapid adaptation during polyploid plant formation. Genome-wide duplication of autotetraploid rice may promote genome-wide hypermethylation of DNA transposons through the RdDM pathway, inhibiting the expression of transposition-related genes to a level similar to that of diploids, and stably promoting polyploid formation [84]. Genomic methylation analysis of synthetic and naturally occurring allotetraploid *Arabidopsis suecica* showed that the differential methylation region remained stable in F1 generation, synthetic and natural tetraploid. This convergent but conserved epigenetic modification may provide the basis for the allotetraploid to stabilize two subgenomes from different species [142]. The study on allotetraploid cotton (*Gossypium hirsutum*) showed that hybridization and polyploidization resulted in the neofunctionalization of transposon-produced lncRNA, which was an important source of increased plant plasticity [143].

In most polyploids, subgenomes can be divided into dominant and recessive subgenomes, and this dominant–recessive relationship is directly related to the distribution of transposons on each subgenome [144]. In *Brassia rapa*, it has been observed that transposon-derived 24-nt siRNA preferentially targets the upstream region of genes located in the recessive subgenome, suggesting that the silenced transposon fragment acts as a *cis*-regulator to regulate gene expression, and the parental genome with the lowest transposon content may become the dominant genome in polyploids [107]. Zhang et al. [109] created a catalogue of genome-wide transcription factor binding sites (TFBSs) in allohexaploid wheat and observed that subgenomic-differentiated TFBSs derived from differential amplification of specific transposons in the diploid ancestor genome, and differential evolutionary selection of transposons promoted the regulation of subgenomic differentiation in wheat.

He et al. [145] constructed a diploid A-genome cotton pan-genome from 344 representative geographic region materials and revealed a contrast pattern of transposon proliferation between diploid and tetraploid cotton, with LTR retrotransposons exhibiting synchronous proliferation in polyploids. In addition, the invasion of LTR retrotransposons from the A subgenome to the D subgenome triggered a large expansion of the latter after polyploidization. This pan-genomic analysis demonstrates the power of pan-genomic approaches in elucidating transposon effects and genomic evolution.

## 10. Future Research Perspectives

Plant transposons are the most variable part of the genome, causing extensive changes in the overall structure and gene function of the genome, affecting the genome regulatory network and evolution, but we have not comprehensively understood the mechanism of plant adaptive evolution caused by transposons. Transposons, together with polyploidy, are considered to be the main drivers of plant genome evolution, but these are not two independent sources of variation, because polyploidy can induce transposon activity, and transposons enable polyploidy to produce new variants [27], so polyploidy speciation is a promising system for studying transposons. Many factors controlling transposon dynamics can be studied, and the variation and evolution of polyploid genomes can be revealed from the perspective of transposon activity, which has made innovative beginnings in recent years [8], but the consequences of hybridization and polyploidy are far more complex than we know.

In particular, many key questions about the role of transposons in polyploid plant genome evolution and their regulatory mechanisms are still worthy of further study, including: (i) Is the success of specific transposon families or specific transposon burst related to specific epigenetic targets or genomic conflicts? (ii) How do transposons trigger silencing, and why does this silencing differ among species? How do transposons escape silencing? (iii) Is there any change in the transposon enrichment area of polyploid plant genomes? Are there differences between different subgenomes? (iv) How do transposons regulate subgenomic gene expression? What are the effects of transposon insertion polymorphism on subgenomic gene expression regulation and dominance? (v) Are plants prone to high levels of transposon-driven changes in stressed and/or unstable environments? etc.

Transposons represent a rich and changing library of genetic and epigenetic variants on which selection can be proceeded. With the development of DNA sequencing technologies, especially the NGS technologies to detect active transposons [146] and long-read sequencing (such as Oxford Nanopore Technologies, ONT) [147], more and more transposons were identified in plant genomes. Accurate assessment of the relative importance of transposons to genome evolution requires a comprehensive analysis of all the sources of genetic and epigenetic variation that work together to contribute to this process, and we currently do not know much about the crucial role that transposons may play. Systematic comparison of a large number of related plant genomes, especially focusing on the effects of genome-wide duplication events on transposon sequences during plant evolution, will provide more evidence for our understanding of the important role of transposons.
